# Development of a Spray-Dried Formulation of Peptide-DNA Nanoparticles into a Dry Powder for Pulmonary Delivery Using Factorial Design

**DOI:** 10.1007/s11095-022-03256-4

**Published:** 2022-04-19

**Authors:** Miftakul Munir, Vicky L. Kett, Nicholas J. Dunne, Helen O. McCarthy

**Affiliations:** 1 School of Pharmacy, Queen’s University Belfast, 97 Lisburn Road, Belfast, BT9 7BL UK; 2 Research and Technology Center for Radioisotope and Radiopharmaceutical, National Research and Innovation Agency, South Tangerang, Indonesia; 3 Centre for Medical Engineering Research, School of Mechanical and Manufacturing Engineering, Dublin City University, Dublin 9, Ireland; 4 Department of Mechanical and Manufacturing Engineering, School of Engineering, Trinity College Dublin, Dublin 2, Ireland; 5 Advanced Manufacturing Research Centre (I-Form), School of Mechanical and Manufacturing Engineering, Dublin City University, Glasnevin, Dublin 9, Ireland; 6 Advanced Materials and Bioengineering Research Centre (AMBER), Royal College of Surgeons in Ireland and Trinity College Dublin, Dublin 2, Ireland; 7 Advanced Processing Technology Research Centre, Dublin City University, Dublin 9, Ireland; 8 Trinity Centre for Biomedical Engineering, Trinity Biomedical Sciences Institute, Trinity College Dublin, Dublin 2, Ireland; 9 School of Mechanical and Manufacturing Engineering, Dublin City University, Dublin 9, Ireland; 10 School of Chemical Sciences, Dublin City University, Dublin 9, Ireland

**Keywords:** cell-penetrating peptide, dry powder formulation, gene delivery, pulmonary delivery, spray drying

## Abstract

**Background:**

Gene therapy via pulmonary delivery holds the potential to treat various lung pathologies. To date, spray drying has been the most promising method to produce inhalable powders. The present study determined the parameters required to spray dry nanoparticles (NPs) that contain the delivery peptide, termed RALA (N-WEARLARALARALARHLARALARALRACEA-C), complexed with plasmid DNA into a dry powder form designed for inhalation.

**Methods:**

The spray drying process was optimised using full factorial design with 19 randomly ordered experiments based on the combination of four parameters and three centre points per block. Specifically, mannitol concentration, inlet temperature, spray rate, and spray frequency were varied to observe their effects on process yield, moisture content, a median of particle size distribution, Z-average, zeta potential, encapsulation efficiency of DNA NPs, and DNA recovery. The impact of mannitol concentration was also examined on the spray-dried NPs and evaluated via biological functionality *in vitro*.

**Results:**

The results demonstrated that mannitol concentration was the strongest variable impacting all responses apart from encapsulation efficiency. All measured responses demonstrated a strong dependency on the experimental variables. Furthermore, spray drying with the optimal variables in combination with a low mannitol concentration (1% and 3%, w/v) produced functional RALA/pDNA NPs.

**Conclusion:**

The optimal parameters have been determined to spray dry RALA/pDNA NPs into an dry powder with excellent biological functionality, which have the potential to be used for gene therapy applications via pulmonary delivery.

## INTRODUCTION

Gene therapy is gaining momentum with more than 3,000 clinical trials approved globally, in which a range of nucleic acids, e.g. ribonucleic acid (RNA), antisense oligonucleotide (ASO), deoxyribonucleic acid (DNA) and delivery systems (e.g. viral, non-viral and physical) have been used across a wide spectrum of pathologies [1]. Key challenges remain that include the degradation of naked nucleic acids by circulating and cytosolic nucleases, and low cellular uptake. Hence, different types of delivery vectors are required to protect and deliver nucleic acids to the target site [2]. This study focusses on a cationic cell-penetrating peptide (CPP) termed RALA, which is a 30 amino acid amphipathic fusogenic peptide (N-WEARLARALARALARHLARALARALRACEA-C). Seven arginines in the RALA’s structure facilitate the condensation of anionic entities, irrespective of size of the nucleic acid. Electrostatic interaction between RALA and such anionic cargo facilitates the assembly of nanosized particles (NPs) [3–5]. The resultant RALA NPs have the size and charge required to enter cells via clathrin mediated processes and escape endosomes through a increased alpha-helicity in a lower pH [6]. This means that the cargo avoids lysosomal degradation and is released at its intracellular destination site [7].

Pulmonary delivery is a promising administration route for treatment of lung pathologies and can mimic a systemic response with exposure to a high lung surface area (~ 142 m^2^) for enhanced drug absorption in addition to providing a non-invasive and painless drug administration route [8, 9]. Despite offering several advantages, pulmonary delivery also poses specific challenges that include: (a) the presence of 23 generations of airway branches, which can result in undesired inhaled particle deposition that reduces drug deposition at the target site, and (b) the ciliated epithelial cells on the inner surface of the lungs that produce mucus for trapping and removal of deposited particles out of the lung. This mucus can also destabilise or reduce the functionality of inhaled NPs and has been reported to thwart more than 25 clinical trials of pulmonary gene therapy [10]. To overcome these challenges, inhalable powders should therefore fall within the desirable range of < 5 µm to reach the lower airways, providing adequate bioavailability to treat lung disease with enhanced absorption for systemic effect. Larger particles typically impact the wall of higher airways, followed by mucociliary clearance and ejection by coughing or swallowing [11]. Another strategy for effective pulmonary delivery is to design inhalable powders that are soluble in mucus or lung surfactant thereby avoiding mucociliary clearance or phagocytosis following mucus trapping [12].

To produce dry powder formulations with the appropriate size for lower airway deposition and desirable solubility to avoid mucociliary clearance, the spray drying method has yielded excellent results. Various nucleic acid and gene vector types have been developed into dry powder form using the spray drying method [13]. To produce physicochemically stable and biologically functional gene-carrying NPs, different formulations and process parameters are needed depending on the characteristics and stability of each individual gene delivery system. For example, liposomes and chitosan-based NPs are stable and functional when spray-dried with an inlet temperature of ≥ 120 °C [14–17], while a lower drying temperature (50–80 °C) is essential for peptide based NPs to avoid thermal degradation [18–20]. Thermal stability during spray drying is also impacted by the excipient that acts as a bulking agent and prevents NP destabilisation due to water loss. Although trehalose has been shown to be an excellent lyophilisation agent for nucleic acids [21], mannitol is the most widely used excipient in the spray drying of nucleic acid NPs because the low hygroscopic nature enables a low drying temperature for dry powder preparation [18–20, 22, 23]. Seven types of CPP-based NPs were spray-dried with a low inlet temperature (50 °C) using mannitol as the excipient, which resulted in stable and functional NPs. Despite using a low inlet temperature, the spray-dried powder demonstrated desirable aerosol performance with a fine particle fraction (FPF) of ~ 50% [20, 24]. Qiu et al. reported the production of PEGylated CPP-based NPs that were also stable when spray dried at a slightly higher temperature (80 °C)*.* These spray-dried NPs demonstrated comparable functionality to commercially available Lipofectamine® 2000 (~ 10^10^ RLU/mg protein). Although the spray drying of CPP-based NPs has been successfully reported, the effects of experimental variables on the conversion of this gene delivery system into dry powder form has not been studied to date. Therefore, the current study is designed to identify the optimal formulation variables and process parameters for spray drying of CPP-based NPs. Specifically, a nano-spray dryer with vibrating mesh for droplet generation and electrostatic separation for product collection was used to produce an inhalable dry powder containing gene-carrying NPs. A full-factorial design methodology was adopted to evaluate four experimental variables and seven responses. The transfection efficiency and cell viability of the resultant spray-dried NPs using optimised process parameters was also investigated.

## MATERIAL AND METHODS

### Materials

The RALA peptide was supplied by Biomatik Corporation (USA). The plasmid encoding Green Fluorescent Protein (pEGFP-N1, 4.7 kb) was purchased from Clontech (USA), propagated in *Escherichia coli* DH5α (Invitrogen, UK) competent cells and extracted by PureLink HiPure Plasmid Maxiprep Kits (Invitrogen, UK). D-mannitol was purchased from Alfa Aesar (UK). DNAse/RNAse free water. Dulbecco’s Modified eagle’s medium (DMEM), and foetal bovine serum (FBS) were purchased from Invitrogen (UK).

### Design of Experiment Methodology

A regular two-factorial design approach was used for optimisation using Design-Expert V12 software (Stat-Ease, USA) to generate 19 randomly ordered experiments based on the combination of four parameters and three centre points per block. One formulation and three process parameters were selected as independent variables. This included mannitol concentration (1–5%), inlet temperature (50–80ºC), spray rate (60–80%), and spray frequency (106–110 kHz). Independent variables and limits were selected based on literature and preliminary studies. The high and low limits of mannitol concentration were selected according to the solubility and the ability to protect NPs upon spray drying. The high limit of inlet temperature was selected by monitoring Z-average and zeta potential to reduce the risk of NP destabilisation. The low limit of inlet temperature was selected by monitoring the dispersibility and moisture content of spray-dried powder. The limits of spray rate and spray frequency were chosen to produce desirable droplet size, minimising the shear stress to NPs and reducing the spray drying time. The measured dependent variables were process yield, moisture content, a median of particle size distribution, Z-average, zeta potential, encapsulation efficiency of DNA NPs, and DNA recovery.

### Preparation of Liquid Formulations

The RALA peptide and pEGFP-N1 were reconstituted separately in DNase/RNase-free water. RALA/pEGFP-N1 NPs were prepared at N:P 10 (which was found to be the optimal N:P ratio) by adding appropriate volumes of peptide solution to pEGFP-N1 solution containing 37.5 μg pDNA, followed by homogenisation and incubation for 30 min at room temperature before further processing. NPs and mannitol solutions were mixed immediately before spray drying.

### Spray Drying

All liquid formulations were spray-dried after preparation by a B-90 nano-spray dryer (BÜCHI Labortechnik, Switzerland) using conditions selected according to the design of experiment study, spray mesh size of 5.5 µm and pump rate of 15% (~ 2.3 mL/min). The liquid formulation was cooled by an ice bath to avoid a temperature rise as a result of liquid circulating through a heated spray head. The humidity inside the drying chamber was reduced by a dehumidifier (BÜCHI Labortechnik, Switzerland). Collected powders were sealed from moisture and stored at 4ºC. The spray-dried powder was stored in a desiccator with silica gel for further analysis. The process yield (%) was subsequently calculated (Eq. 1)

1 $$Process\;yield=\frac{Collected\;powder\;mass}{Initial\;total\;dry\;mass}\times100\%$$

### Characterisation of Microparticles

#### Particle Size Distribution

Particle size distribution of the spray-dried formulation was measured using a HELOS/BR Laser Light Diffraction Analyser (Sympatec, Germany) with R2 lens. Approximately 1 mg of spray-dried powder was suspended in chloroform in a 50 mL glass cuvette and was stirred with a magnetic bar at 1,000 RPM. Subsequently, the mixture was sonicated at a power of 60 W for 60 s (CUVETTE, 8.5 mm diameter ultrasound tip; Sympatec, Germany) prior to measurement.

#### Moisture Content

Moisture content was measured by Q50 Thermo-Gravimetric Analysis (TGA) (TA Instruments, USA). Approximately 1 mg of spray-dried powder was placed into an aluminium standard pan (TA Instruments, USA), followed by heating from 0–150ºC at a rate of 10ºC/min. Universal analysis software (TA Instruments, USA) was used for thermal data analysis.

#### Scanning Electron Microscopy

For scanning electron microscopy (SEM), the spray-dried powder was placed onto sticky carbon tape mounted on SEM stubs and observation was conducted using a field emission TM3030 scanning electron microscope (Hitachi, Japan) at different magnifications (i.e., × 1000 and × 4000).

#### Differential Scanning Calorimetry

Differential scanning calorimetry (DSC) analysis was performed on Q50 DSC (TA Instruments, USA). Approximately 1 mg of spray-dried powder was sealed into a hermetic aluminium pan (TA Instruments, USA), and subsequently heated from 25–200ºC at a heat rate of 5ºC/min. Universal analysis software (TA Instruments, USA) was used for thermal data analysis.

### Characterisation of Nanoparticles

#### Size and Charge of Nanoparticles

Z-average size and zeta potential of spray-dried NPs were determined by dynamic light scattering and laser Doppler velocimetry, respectively, using a Malvern Nano ZS Instrument with DTS software (Malvern Instruments, UK). An appropriate amount of spray-dried powder containing 0.5 µg pEGFP-N1 was dissolved in 50 µL DNase/RNase-free water. Z-average was determined by transferring 50 µL of solution into a polystyrene cuvette (Starstedt, UK), while zeta potential was determined by diluting 50 µL of the solution to 1 mL using DNase/RNase-free water in a folded capillary cuvettes (Malvern Instruments, UK). 70% ethanol and DNase/RNase-free water were used to wash the cuvettes before sample loading.

#### Particle Size Distribution

Z-average size and zeta potential of NPs were determined by dynamic light scattering and laser Doppler velocimetry using a Malvern Nano ZS Instrument with DTS software (Malvern Instruments, UK). Spray-dried powder containing 0.5 µg pEGFP-N1 was reconstituted in 50 µL DNase/RNase-free water and transferred into a polystyrene cuvette (Starstedt, UK) for Z-average measurement. Zeta potential was measured by diluting 50 µL of the reconstituted solution to 1 mL using DNase/RNase-free water in a folded capillary cuvette (Malvern Instruments, UK). 70% ethanol and DNase/RNase-free water was used to wash the cuvettes before sample loading.

#### Encapsulation Efficiency

The encapsulation efficiency of NPs was determined by measuring the free pEGFP-N1 complexed by PicoGreen assay. 50 µL of reconstituted spray-dried powder and DNA control were transferred to 96 well black plates. 50 µL of Quant-iT™ PicoGreen dsDNA reagent (Invitrogen, UK) at a concentration of 0.5% in TAE buffer was added to each sample. The fluorescence intensity was analysed with an excitation wavelength of 480 nm and an emission wavelength of 520 nm on a FLUOstar plate reader (BioTek Instruments Inc., UK). Encapsulation efficiency (%) was determined using Eq. 2.

2 $$Encapsulation\;efficiency=\left(\frac{{\varvec{T}}{\varvec{o}}{\varvec{t}}{\varvec{a}}{\varvec{l}}\boldsymbol\;{ }{\varvec{D}}{\varvec{N}}{\varvec{A}}\boldsymbol{ }\boldsymbol{ }-\boldsymbol{ }{\varvec{f}}{\varvec{r}}{\varvec{e}}{\varvec{e}}\boldsymbol\;{ }{\varvec{D}}{\varvec{N}}{\varvec{A}}}{{\varvec{T}}{\varvec{o}}{\varvec{t}}{\varvec{a}}{\varvec{l}}\boldsymbol\;{ }{\varvec{D}}{\varvec{N}}{\varvec{A}}\boldsymbol{ }}\right)\times 100\boldsymbol{\%}$$

#### DNA Recovery

DNA recovery from spray-dried powder was performed by DNA quantification method that has been reported elsewhere with a modification [26]. Briefly, 50 µL of the reconstituted spray-dried powder and different concentrations of RALA/pDNA as standard curves were transferred to 96 well black plates. 50 µL of proteinase K (BioUltra, ≥ 30 units/mg protein) at a 1 mg/mL concentration in DNase/RNase-free water was added to each sample and incubated for 90 min at 37ºC. 50 µL of Quant-iT™ PicoGreen dsDNA reagent at a concentration of 0.5% was then added to each sample and incubated for 30 min. The fluorescence intensity was analysed with an excitation wavelength of 480 nm and an emission wavelength of 520 nm using a FLUOstar plate reader (BioTek Instruments Inc., UK). DNA recovery from spray-dried powder was determined by plotting the fluorescence in standard curves.

### Cell Culture

A549 (human lung adenocarcinoma epithelial cells) was purchased from ATCC (USA). The cells were maintained at 5% CO_2_ and 37 °C in DMEM supplemented with 10% FBS. The cells were subcultured twice weekly and were routinely confirmed to be Mycoplasma free.

### *In Vitro* Transfection Study

A549 cells were seeded to 96 well plates at a density of 1 × 10^4^ cells/well, and incubated overnight. The media was replaced with 100 µL Opti-MEM serum free-media for 2 h before transfection. Spray-dried powder equal to 0.5 µg DNA, freshly prepared NPs, or DNA control was added to cells. After 4–6 h of incubation at 37 °C, the media was replaced with 200 µL of DMEM. The green fluorescence protein (GFP) expression in the cells was then measured using fluorescent microscopy and flow cytometric analysis 48 h later. To calculate transfection efficiency, GFP positive cells were calculated out of 10,000 collected events per run via FACs analysis. This was then converted into a % transfection.

### Fluorescent Microscopy

Fluorescent images of transfected A549 cells were observed 48 h post-transfection using a 240 Nikon Eclipse TE300 inverted microscope with epifluorescence attachment (Nikon, USA) and images were captured using a Nikon DXM1200 digital camera (Nikon, USA) at a × 200 magnification.

### Flow Cytometric Analysis

At 48 h post-transfection, the media was removed and washed with PBS. Subsequently, cells were trypsinised and resuspended in FACS buffer prior to immediate analysis using a FACS-Calibur system (BD Biosciences, UK) with 10,000 collected events. The suspension of untreated cells was used for a gate setting of 0.7%, and gating was used to remove cell debris, doublet cells, and non-GFP fluorescence.

### Cell Viability Study

A549 cells were transfected, as detailed in the *in vitro* transfection study section. At 48 h post-transfection, 10 µL Alamar blue cell viability reagent (Invitrogen, UK) was added to the cells and incubated in the dark. After 2–3 h, 50 µL of cell’s media was transferred into a black plate and the sample fluorescence was analysed with an excitation wavelength of 560 nm and a emission wavelength of 590 nm using a FLUOstar plate reader. Cell viability was calculated as a relative percentage to the untreated control and ISO 10993–5 was used as the standard method. Categorisation was ranked as weakly (80–60%), moderately (60–40%) or strongly cytotoxic (< 40%).

### Statistical Analysis

Statistical analysis was performed using Prism 8 (GraphPad Software, USA). All data was obtained from three independent experiments and reported as mean values ± SEM, unless otherwise specified.

## RESULTS AND DISCUSSION

### Design of Experiment

The list of experiments randomly formulated using Design-Expert software and the results of seven measured responses are presented in Table I.

**Table I:** Full Factorial Design of Experimental Variables and Values for Measured Responses

Run	Design				Responses						
	MC	IT	SR	SF	PY	MCT	MD	ZA	ZP	EE	DR
1	1	50	80	110	45	1	5	212	33	83	85
2	1	80	80	106	31	0.7	3	202	28	79	86
3	3	65	70	108	68	0.9	3	208	22	59	80
4	5	50	60	106	77	0.8	3	265	25	66	77
5	1	50	80	106	47	1	2	285	36	81	78
6	5	80	80	110	82	0.9	3	179	18	79	77
7	1	80	60	106	35	0.9	4	307	23	70	82
8	5	80	60	110	71	0.7	3	155	15	76	77
9	5	50	80	110	66	0.8	3	170	24	80	83
10	1	80	60	110	36	0.7	13	171	27	78	87
11	1	50	60	106	42	1	5	156	32	81	83
12	3	65	70	108	62	0.7	2	174	25	83	71
13	1	80	80	110	50	0.8	11	140	29	78	88
14	5	50	80	106	53	0.9	3	157	21	82	77
15	5	50	60	110	82	0.7	3	209	23	73	77
16	5	80	60	106	69	0.7	2	169	24	71	75
17	5	80	80	106	69	0.8	3	179	18	73	77
18	1	50	60	110	63	0.9	7	197	25	60	81
19	3	65	70	108	54	1	5	197	31	25	60

#### Design of Experiments Randomly Generated by Design Expert 12

(MC) mannitol concentration (%), (IT) inlet temperature (ºC), (SR) spray rate (%), (SF) spray frequency (kHz), (PY) process yield (%), (MCT) moisture content (%), (MD) median diameter (µm), (ZA) Z-average (nm), (ZP) zeta potential (mV), (EE) encapsulation efficiency (%), (DR) DNA recovery (%).

#### Process Yield

**Fig. 1:**
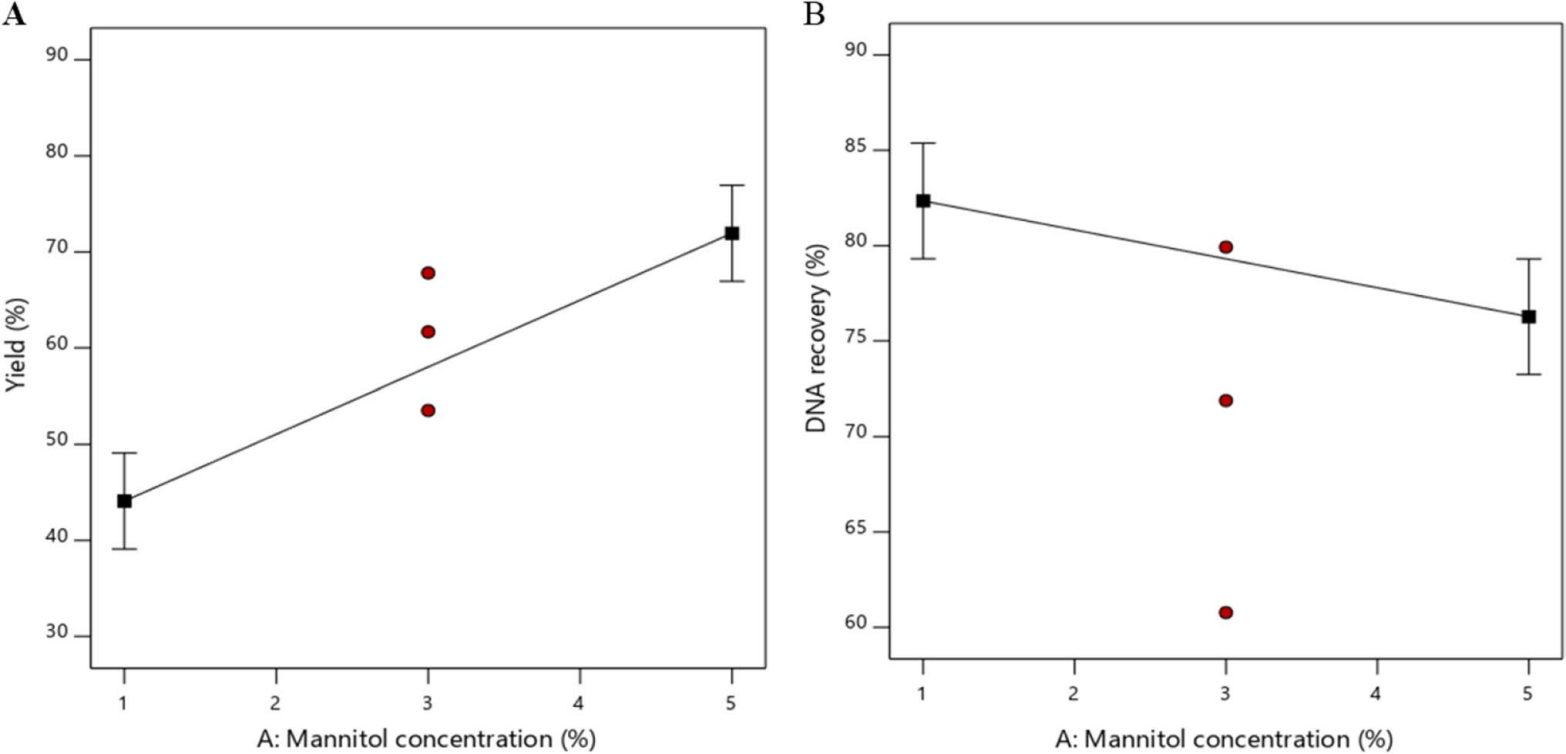
One Factor Plot Representing the Influence of (**A**) Mannitol Concentration on Process Yield, (**B**) Mannitol Concentration on DNA Recovery

Process yield is considered an important parameter in inhalable drug development and production, particularly for valuable and expensive biomaterials such as nucleic acids. The process yield (Fig. 1A) was significantly influenced only by mannitol concentration (p < 0.0001). An increase in the mannitol concentration from 1 to 5% (w/v) significantly increased the product yield. It was visually observed that a similar amount of powder was left inside the drying chamber or collecting electrode irrespective of the mannitol concentration, resulting in a higher process yield at a higher total dry mass of a sample. A similar finding was reported by Abdel-Mageed et al., where α-amilase was spray-dried by an instrument with vibrating mesh spray head and electrostatic product collection. Abdel-Mageed et al. found that excipient type and mesh size significantly affected the process yield due to the influence on the powder stickiness, influencing the loss on chamber wall and collecting electrode [27]. Draheim et al. suggested that the solvent type indirectly determines the electrostatic charge in powder, essential for the product precipitation on the collecting electrode [28]. Taken together, these studies suggest that powder loss was typically caused by wall deposition and loss during manual collection, controlled by excipient type and concentration, mesh size, and solvent type [27–29]. All parameters mentioned above were fixed in this study except for excipient concentration, therefore only one significant independent variable was found.

The optimisation of process yield using another spray dryer type shows different results due to different spray head and collection systems. Mohajel et al*.* reported feed rate as the most significant factor determining process yield since it influenced the moisture content of the resulting powder [17]. This finding can be explained by the fact that the cyclone-based spray dryer poses tortuous flow from the main chamber to product collector, resulting in a higher probability of wall deposition in case the powder has a high moisture content. In contrast, a nano-spray dryer with a spray head and collector installed in a single chamber exhibits less wall deposition [30].

#### DNA Recovery

DNA recovery has been reported as an important parameter in the spray drying of gene-carrying NPs since the presence of various physical stresses, such as shear and thermal stress, affects nucleic acid stability [17, 31]. DNA recovery after spray drying was found at a range of 61–89%, and mannitol concentration had a significant effect on DNA recovery (p < 0.05), (Fig. 1B). The negative effect of mannitol concentration on DNA recovery was related to rapid crystallisation of mannitol, resulting in NP destabilisation [32, 33]. This destabilisation will reduce the ability of NPs to protect nucleic acid cargo from degradation [17]. Nucleic acid cargo degradation post spray drying can also be induced by shear stress during droplet generation in a gas atomiser [17]. However, droplet generation by vibrating mesh with medium mesh size (5.5 µm) and spray frequency (106–110 kHz) in this experiment demonstrates lower shear stress as proven by maintained DNA recovery [34, 35]. The challenge in the vibrating mesh-based spray dryer is a longer operation time that can prolong hot air exposure to the collected powder inside the drying chamber. Therefore relatively low inlet temperatures were used (50–80 °C) to reduce the thermal stress.

#### Particle Size Distribution

**Fig. 2:**
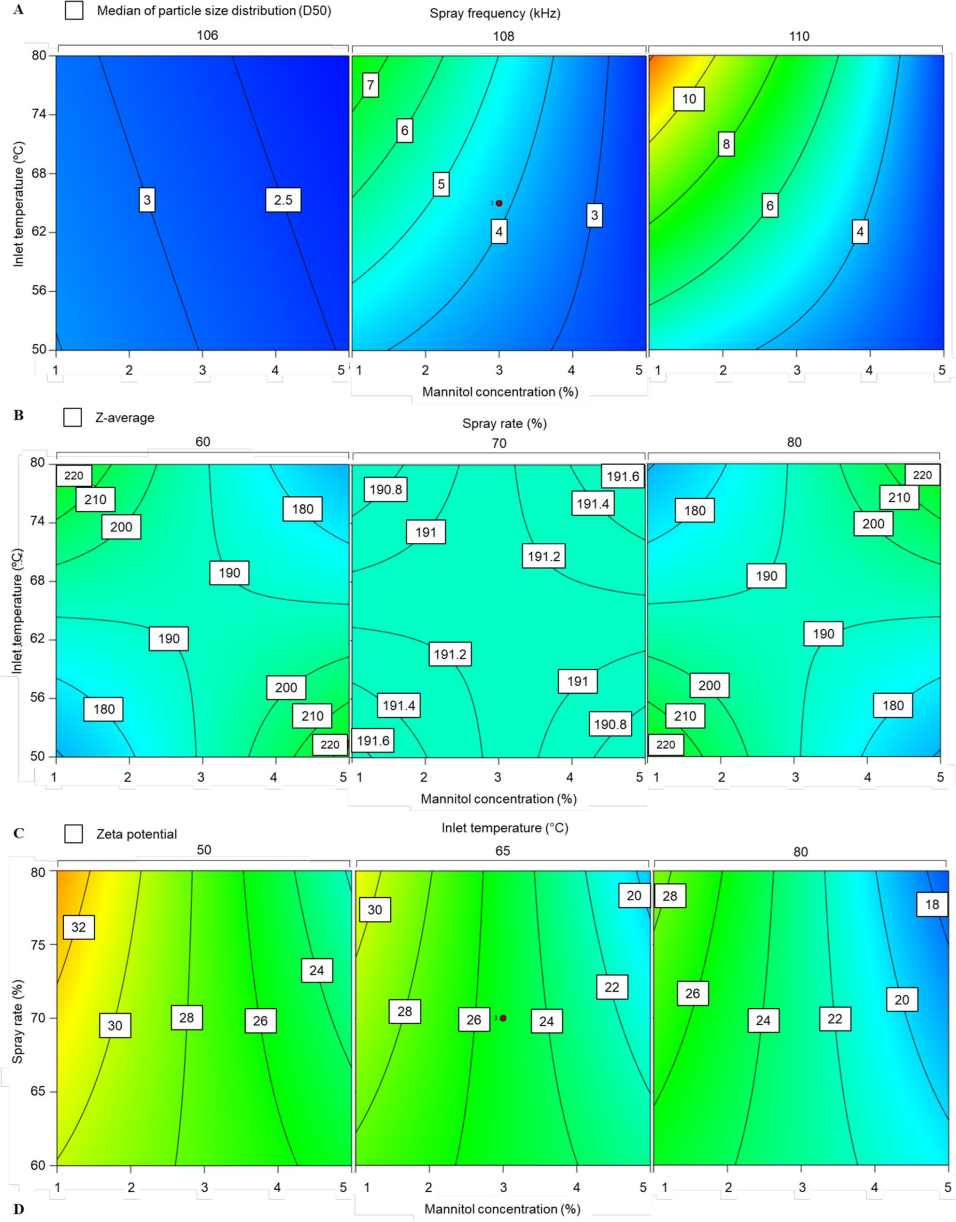
Contour Plots Presenting a Variation of (**A**) Median of Particle size Distribution Measured by Laser Light Diffraction Analyzer in Response to Spray Inlet Temperature, Mannitol Concentration and Spray Frequency; (**B**) Z-average in Response to Inlet Temperature, Mannitol Concentration, and Spray Rate; (**C**) Zeta Potential in Response to Inlet Temperature, Mannitol Concentration, and Spray Rate

The particle size distribution measurement is essential due to the significant influence on the lung deposition of inhalable powder, which eventually determines the therapeutic effect of drug delivered via pulmonary delivery. An undesired lung deposition can result in adverse effects or low bioavailability [9, 36]. It has been reported that the ideal particle size of the inhalable powder is < 5 µm to avoid powder deposition in the upper airway wall, followed by mucociliary clearance [37]. In the optimisation process, particle size distribution was presented as a median of particle size distribution (D_50_) and ranged from approximately 2 to 13 µm (Table I). Only four experiments with mannitol concentration of 1% resulted in a D_50_ > 5 µm, which implies the strong effect of mannitol concentration on D_50_. A contour plot generated by Design Expert demonstrates that the lowest moisture content could be achieved when mannitol concentration and spray frequency were at the highest limit while inlet temperature was minimal (Fig. 2A). Statistical analysis showed that all independent variables were found to significantly influence D_50_ except for the spray rate (Table II). As previously predicted, mannitol concentration had the most significant decreasing effect out of all the variables (p < 0.0001). In addition, the interaction of inlet temperature, mannitol concentration, and spray frequency also had a reducing effect (0.0061), and the interaction between mannitol concentration and spray frequency had the most significant increasing effect (p** < **0.001).

**Table II:** Statistically Significant Experimental Variables for the Spray Drying of RALA/pEGFP-N1 NPs

Response	Variable	P-value
Yield	MC	< 0.0001
Moisture content	MC	< 0.05
	IT	< 0.05
	MC-IT	< 0.05
Median diameter	MC	< 0.0001
	IT	< 0.001
	SF	< 0.001
	MC-IT	< 0.01
	MC-SF	< 0.001
	IT-SF	< 0.01
	MC-IT-SF	< 0.01
Z-average	MC-IT-SR	< 0.01
Zeta potential	MC	< 0.0001
	IT	< 0.01
	MC-SR	< 0.05
	MC-IT-SF	< 0.05
DNA recovery	MC	< 0.05

A p-value of < 0.05 (ANOVA) was considered to be statistically significant. Variables are labelled with a single letter for simplification: mannitol concentration (MC), inlet temperature (IT), spray rate (SR), and spray frequency (SF).

It has been reported that excipient concentration typically increases D_50_ upon spray drying [17, 25]. Mohajel et al. suggested the equation developed by Nukiyama and Tansawa (Eq. 3) to explain this finding [17].

3 $${D}_{particle}\cong {D}_{droplet}\times \sqrt[3]{C\times \frac{{\rho }_{droplet}}{{\rho }_{particle}}}$$

where D is the diameter of droplet or particle, C is the concentration of solid content in the sample, and ρ is the density of droplet or particle. Equation 3 suggests the increasing effect of excipient concentration on D_50_. The different finding in this experiment was probably caused by low inlet temperatures (50–80ºC) and the absence of dispersibility enhancers, resulting in a higher chance of aggregation in the low mannitol concentration with higher water composition. As a result, a larger particle size was observed in lower solid concentrations as a result of aggregation. Nevertheless, the majority of D_50_ in our study was < 5 µm, which falls within the desired size range of the inhalable powder [37].

The significant decreasing effect of inlet temperature on D_50_ can be explained by droplet shrinkage during the drying process. For higher temperatures, the shrinkage of a mannitol-based droplet is typically enhanced, providing a smaller dry powder [31]. However, this effect was the weakest (p = 0.0081) compared to other independent variables since the inlet temperature range was relatively narrow (50–80ºC). The decreasing effect of spray frequency (p = 0.0003) was found to be stronger than that of inlet temperature since spray frequency directly determined the size of the droplet, and droplet size relates to dry particle size (Eq. 3). Aside from mesh size, the spray frequency significantly influences droplet size, where a higher vibration frequency generates smaller droplets due to more droplet production [38].

#### Z-average

The Z-average of the spray-dried RALA/pEGFP-N1 NPs ranged from approximately 140 to 307 nm (Table I), which is larger than the freshly prepared NPs (82 ± 8 nm). A Z-average of < 200 nm is required for effective cellular uptake as RALA/pEGFP-N1 NPs have been reported to penetrate cellular membrane via clathrin- or caveolin-mediated endocytosis pathway [5, 39]. Although several experiments resulted in a Z-average of > 200 nm, the majority of Z-average value (12 experiments) were < 200 nm. The statistical analysis showed that only the interaction of inlet temperature, mannitol concentration, and spray rate significantly affected Z-average (p < 0.01). This finding differs from the study reported by Mohajel et al., where spray air flow rate and inlet temperature were found to be the variables that significantly affected the Z-average [17]. Mohajel et al*.* used a gas atomiser spray dryer, in which spray airflow played an important role in maintaining NP stability as it utilised air to split the sample fluid into tiny droplets, resulting in shear stress that affected NP stability [40]. On the contrary, the vibrating mesh spray head in our study only utilised mesh vibration for droplet generation, where spray frequency controlling mesh vibration in the spray head is a more important parameter for NP stability. Nevertheless, spray frequency was not a significant variable in our study since the range values resulted in acceptable vibration to maintain minimum shear stress. A failure in revealing inlet temperature as a single effect on Z-average can be due to the narrow gap between a low and high limit [41]. Such a narrow gap was applied to protect NPs and produce a dispersible powder. Applying an inlet temperature of > 80ºC in the spray drying of CPP-based NP can severely reduce the NP stability, while a temperature < 50ºC will result in clumped powder [18–20].

The combination effect of inlet temperature, mannitol concentration, and spray rate on NP stability during drying using nano-spray dryer has not been studied before. However, this effect might be due to the crystallisation process during droplet drying that destabilises the NPs [32]. It has been reported that mannitol crystallisation behaviour (i.e. crystal size and form) was influenced by concentration, temperature, and droplet size [42]. Although there is no study investigating the influence of crystallisation behaviour on the Z-average during spray drying, our finding suggests the negative effect of mannitol crystallisation in higher concentration and temperature and smaller droplets on NP stability, reducing the zeta potential. The effect of inlet temperature, mannitol concentration and spray rate on Z-average is presented as a contour plot (Fig. 2B). The minimum Z-average could be obtained in four different regions: (1) the inlet temperature, mannitol concentration and spray rate are minimum, (2) the inlet temperature was minimum, mannitol concentration and spray rate were maximum, (3) mannitol concentration was minimum, inlet temperature and spray rate were maximum and (4) spray rate was minimum, inlet temperature and mannitol concentration were maximum.

#### Zeta Potential

The zeta potential of spray-dried RALA/pEGFP-N1 NPs ranged from 18 to 3**7** mV (Table I), which is advantageous for effective cellular uptake (positive charge). Effective penetration across a cellular membrane is typically performed by positively charged NPs since it has the ability to interact with the phosphate groups of the membrane [5, 39, 43]. Furthermore, electrostatic repulsive forces provided by the positive charged can help prevent the formation of NP aggregates [44]. Statistical analysis demonstrated that zeta potential was significantly influenced by inlet temperature (p** < **0.01) and mannitol concentration (p < 0.0001) (Table II). Similar to Z-average, this effect is most likely due to mannitol crystallisation controlled by drying temperature and mannitol concentration [33]. Furthermore, higher inlet temperature also posed thermal stress, affecting NP stability, eventually reducing zeta potential [32]. Despite being negatively affected by inlet temperature and mannitol concentration, all the measured zeta potentials were sufficient for effective cellular uptake [39]. The interaction of mannitol concentration and spray rate also demonstrated a significant negative effect on zeta potential (p** < **0.05), which can be explained by the increase of shear stress due to the interrelationship between both variables. Arultmuthu et al. suggested that shear level in droplet generation by vibrating mesh is determined by the ability of a molecule to pass through the mesh hole [35, 45]. A higher mannitol concentration increases solid content passing through the mesh hole, and spray rate increase feed solution onto vibrating mesh, which simultaneously increases the shear stress on NPs. The interaction of inlet temperature, mannitol concentration, and spray frequency also negatively affected zeta potential (p** < **0.05). Similar to the individual effect of inlet temperature and mannitol concentration, this combination effect was likely due to the crystallisation effect on NP stability, given the fact that mannitol is easy to crystallise during the drying process. The variation of zeta potential in response to inlet temperature, mannitol concentration, and spray rate were reported to be sufficient for effective cellular uptake (Fig. 2C).

#### Moisture Content

**Fig. 3:**
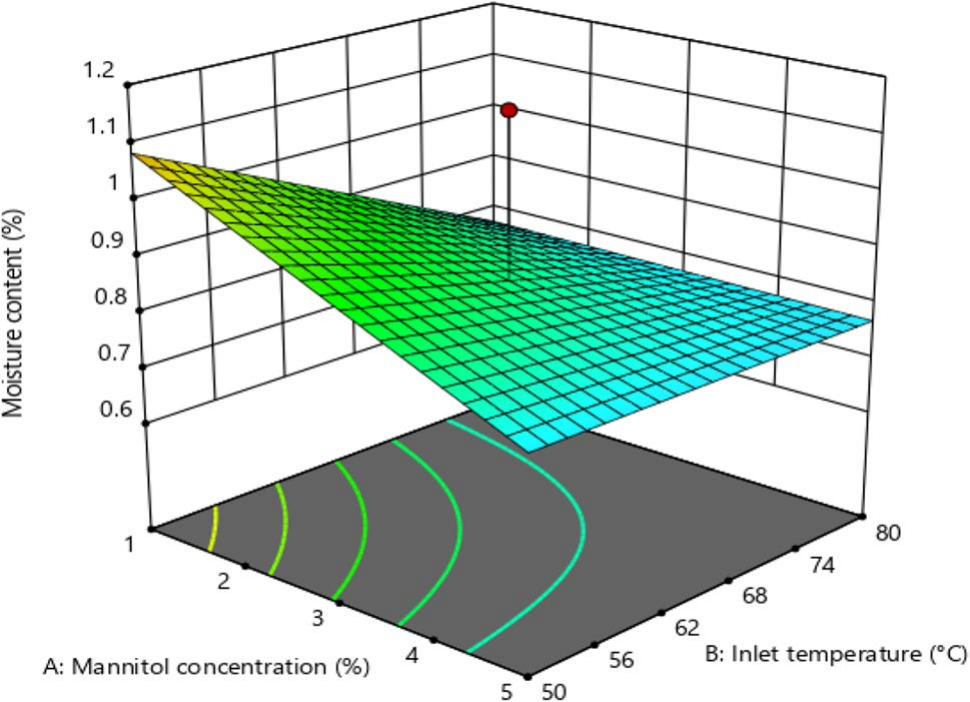
3D Plot Representing the Effect of the Mannitol Concentration and Inlet Temperature on Relative Moisture Content

Moisture content is an essential parameter in inhalable powder as it determines the aerosol performance. Low moisture content is required to avoid powder aggregation and chemical, microbial, and physical degradation upon long-term storage [29]. The lowest moisture content was achievable at the maximum point of inlet temperature and mannitol concentration (Fig. 3). Inlet temperature (p** < **0.05) and mannitol concentration (p** < **0.05) were two main variables that significantly influenced the moisture content of the spray-dried powder. The interaction of both variables showed a weaker effect on moisture content (p** < **0.05). Increasing inlet temperature resulted in a reduction in the relative humidity of drying gas and an increase in solvent evaporation, eventually resulting in lower moisture content of dry powder [29, 46]. Low moisture content can simply be achieved by applying high temperature. However, the inlet temperature should be kept below the point (80ºC), where the NPs may be damaged [18–20, 47]. Mannitol concentration also influences the moisture content of dry powder since it determines the amount of solvent in a droplet, in which a high mannitol concentration produces droplets with higher solid and lower solvent composition, resulting in lower moisture content in dry powder [29]. The mechanism of the effect of the inlet temperature ratio in reducing moisture content via interaction with mannitol concentration is related to the effectiveness of solvent evaporation from droplets affected by the solvent amount. As discussed earlier, the solvent amount in the droplet is determined by mannitol concentration.

#### Encapsulation Efficiency

Encapsulation efficiency represents the amount of pDNA condensed in the RALA NP. The measured encapsulation efficiency ranged from 25 to 83% (Table I), and no independent variable significantly influenced the encapsulation efficiency (Table II). Most of the encapsulation efficiency values were > 65%, and few observed low values were predicted due to experimental error. This finding implied that RALA NP can still effectively condense pDNA even though NP stability was reduced by different conditions (e.g. crystallisation, shear and thermal stress), which was demonstrated by an increase in Z-average and a decrease in zeta potential. Dormenval et al*.* reported the encapsulation efficiency of lipidoid polymer hybrid/siRNA NPs ranged from 32–59% after spray drying with a gas atomiser and cyclone separator-based instrument [46]. It could be suggested that such a spray dryer type demonstrates higher physical stress affecting encapsulation efficiency or that the lipidoid polymer hybrid vector did not effectively condense siRNA, resulting in more susceptibility to physical stress.

### Optimised Process Parameter

This DoE aspired to achieve dispersible powder containing RALA/pEGFP-N1 NPs with retained functionality. However, the transfection study was not involved in the response list of DoE due to cost efficiency. Instead, other important parameters in cellular uptake and transfection efficiency, e.g. Z-average and zeta potential, were included [5, 39]. Nevertheless, although DoE can statistically generate optimised experimental variables to produce optimal Z-average and zeta potential, the sugar concentration used in spray drying can still affect the cellular uptake and gene expression, depending on the concentration or osmolarity, which impact the transfection efficiency [48, 49]. Liang et al*.* reported that spray-dried LH4-L1/pDNA NPs with 1% mannitol demonstrated sevenfold higher transfection efficiency in A549 cells than the freshly prepared NPs (10^4^–10^5^ RLU/mg protein) [20]. In contrast, Huang et al*.* reported that the presence of 0.45 M sucrose reduced the cellular uptake of fluorescein isothiocyanate chitosan NPs in A549 cells owing to the inhibition of the clathrin-mediated endocytosis pathway [50]. Therefore, mannitol concentration was set in low, middle, and high levels (2, 3, and 5%) to assess the influence on NPs functionality. The experimental variables optimised by Design-Expert V12 for the spray drying of RALA/pEGFP-N1 NPs were inlet temperature of 50 °C, spray rate of 80%, and spray frequency of 110 kHz. Aside from producing spray-dried NPs with maintained functionality, those optimised experimental variables were also generated to produce inhalable RALA/pEGFP-N1 with high product yield, low moisture content, D_50_ from 1 µm to 5 µm, Z-average of < 200 nm, positive zeta potential, encapsulation efficiency of > 75%, and DNA recovery of > 75%. The result of spray drying using an optimised experimental variable is presented in Table III as ‘confirmation run’ along with the predicted response generated by Design-Expert software. All three different mannitol concentrations resulted in product yield of ≥ 70%, moisture content of ≤ 1%, D_50_ from 3.05 µm to 3.18 µm (the accurate size distribution data are given in Table IV), Z-average of ≤ 167 nm, zeta potential from 21 to 28 mV, encapsulation efficiency of ≥ 79%, and DNA recovery of ≥ 78%. The Design-Expert software also generated ‘desirability’, which is the possibility to produce a spray-dried powder with the desired characteristics using the optimised experimental variables. The desirability of 0.7–0.8 explains the slight difference between conformation run and predicted responses. Product yield, encapsulation efficiency, and DNA recovery typically showed higher confirmation response than the predicted one, which is beneficial in term of cost value and NP quality. In contrast, D_50_ and Z-average typically showed lower confirmation response than the predicted one, which is advantageous in term of inhalable powder and NP quality. Although the lower confirmation response of zeta potential does not enhance the NP quality, such a decrease also did not reduce the NP quality since the zeta potential remains within the desired value (> 21 mV) [39, 43]. All responses of 5% mannitol, except for D_50_, and zeta potential, demonstrated closer confirmation and predicted response values compared to other mannitol concentrations, which is due to the higher desirability of 5% mannitol. The discrepancy in D_50_ and zeta potential might be owing to the high number of parameter interactions affecting these responses (Table II). With such parameter interactions, slight changes in spray dryer performance, e.g. gas flow and chamber pressure, can alter the value of D_50_ and zeta potential.

**Table III:** A comparison between the predicted responses by Design Expert 12 using the optimised experimental variables and the final ‘confirmation run’

Spray-dried RALA/pEGFP-N1		Yield (%)	Moisture content (%)	D_50_ (µm)	Z-average (nm)	Zeta potential (mV)	Encapsulation efficiency (%)	DNA recovery (%)	Desirability
2% mannitol	Pred	50.71	1.0	4.3	199.1	29.7	72.4	80.9	0.72
	Con	74.4 ± 7.2	0.7 ± 0.1	3.2 ± 0.8	163.7 ± 8.8	28.0 ± 0.8	85.3 ± 0.0	78.0 ± 0.0	
3% mannitol	Pred	58.0	0.9	3.7	185.7	27.5	72.4	79.3	0.79
	Con	69.9 ± 3.7	1.1 ± 0.1	3.2 ± 0.1	152.3 ± 1.9	21.3 ± 1.6	80.3 ± 0.0	81.7 ± 0.0	
5% mannitol	Pred	71.9	0.8	2.4	165.8	23.4	76.2	76.3	0.83
	Con	71.5 ± 4.0	0.8 ± 0.1	3.1 ± 0.3	167.2 ± 17.2	25.1 ± 1.0	79.5 ± 1.0	80.8 ± 0.9	

Pred: predicted responses; Con: confirmation run.

**Table IV:** Particle Size Distribution of Spray-Dried RALA/pEGFP-N1 NPs. Approximately 1 mg of spray-dried RALA/pEGFP-N1 NPs were suspended in chloroform in a 50 ml glass cuvette and were stirred with a magnetic bar at 1000 rpm. Subsequently, a 60 s of sonication at a power of 60 W was performed before measurement

Spray-dried RALA NPs	D_10_ (µm)	D_50_ (µm)	D_90_ (µm)	Span
2% mannitol	1.56 ± 0.34	3.18 ± 0.78	7.85 ± 1.95	2.05 ± 0.55
3% mannitol	1.20 ± 0.40	3.17 ± 0.06	7.03 ± 0.94	1.85 ± 0.34
5% mannitol	1.46 ± 0.05	3.05 ± 0.31	8.04 ± 2.45	2.24 ± 1.01

Dx: particle size distribution at x% of the volume distribution; Span, width of the volume distribution relative to the median diameter (D_90_—D_10_)/D_50_.

### Powder Properties

#### Scanning Electron Microscopy

**Fig. 4:**
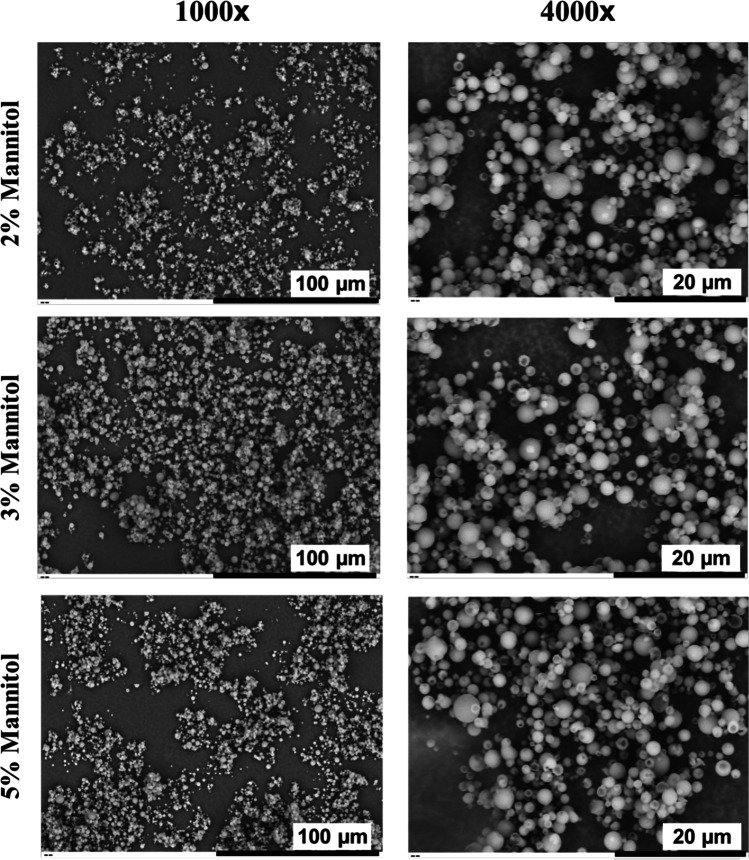
Micrographs of Spray-Dried RALA/pEGFP-N1 NPs as a Function of Mannitol Concentration. Spray-Dried RALA/pEGFP-N1 NPs were Sprinkled onto Sticky Carbon Tape Mounted on SEM Stubs and were Observed using SEM at Magnifications of 1000 × and 4000x

To assess the morphology of spray-dried RALA/pEGFP-N1 NPs, SEM observation was carried out. SEM micrographs of the spray-dried RALA/pEGFP-N1 NPs at three different mannitol concentrations exhibited NP of similar particle dispersibility and sphericity (Fig. 4). All samples were dispersible with slight aggregation and spherical, which is typically generated in the spray drying of mannitol at a low inlet temperature. The main advantage of using mannitol as an excipient is the ability to produce low moisture content even in low-temperature drying, which is the most important parameter to yield dispersible powder [19, 20, 22]. The spherical particle is also expected in this spray drying condition since a highly saturated coating or hard outer layer is formed due to crystallisation after water loss at the droplet surface, followed by slow evaporation of the remaining water in the core through the solid outer shell. In contrast, at a high temperature, water evaporation is faster than crystallisation in the droplet surface, preventing the formation of a solid outer layer and leading to a non-spherical shape [51].

Another characteristic typically assessed using SEM analysis is particle smoothness. However, the SEM magnification (Fig. 4) was not sufficient to thoroughly observe the particle smoothness. Nevertheless, spray drying of mannitol in a low inlet temperature typically produces particles with a smooth surface due to the slow crystallisation process, resulting in fine needles. A corrugated surface is typically formed at high-temperature drying owing to fast crystallisation, resulting in larger and coarse crystals [52]. Due to the ability to prevent particle aggregation, a corrugated surface is typically more beneficial to aerosol performance compared to a smooth surface [53]. Chew et al*.* suggested that reduced particle aggregation is most likely due to lower van der Waals force in the corrugated powder. This surface type can prevent close contact among particles, resulting in a lower van der Waals force (Eq. 3) [54].

4 $${F}_{vdw}=\frac{A\times R}{{12H}^{2}}$$

where F_vdw_ is van der Waals force, A is the Hamaker constant, R is particle radius, and H is inter particulate distance. Chew et al*.* also suggested that van der Waals force is also reduced by the lower inter particulate contact area in the corrugated surface [54]. Although higher temperature drying and the addition of leucine can generate corrugated surface particles, it can also reduce NP stability and functionality [15]. Therefore, the addition of another compound in spray drying formula, e.g. trimethyl chitosan [14], can be investigated to improve surface roughness while retaining NP stability and functionality.

#### Differential Scanning Calorimetry

**Fig. 5:**
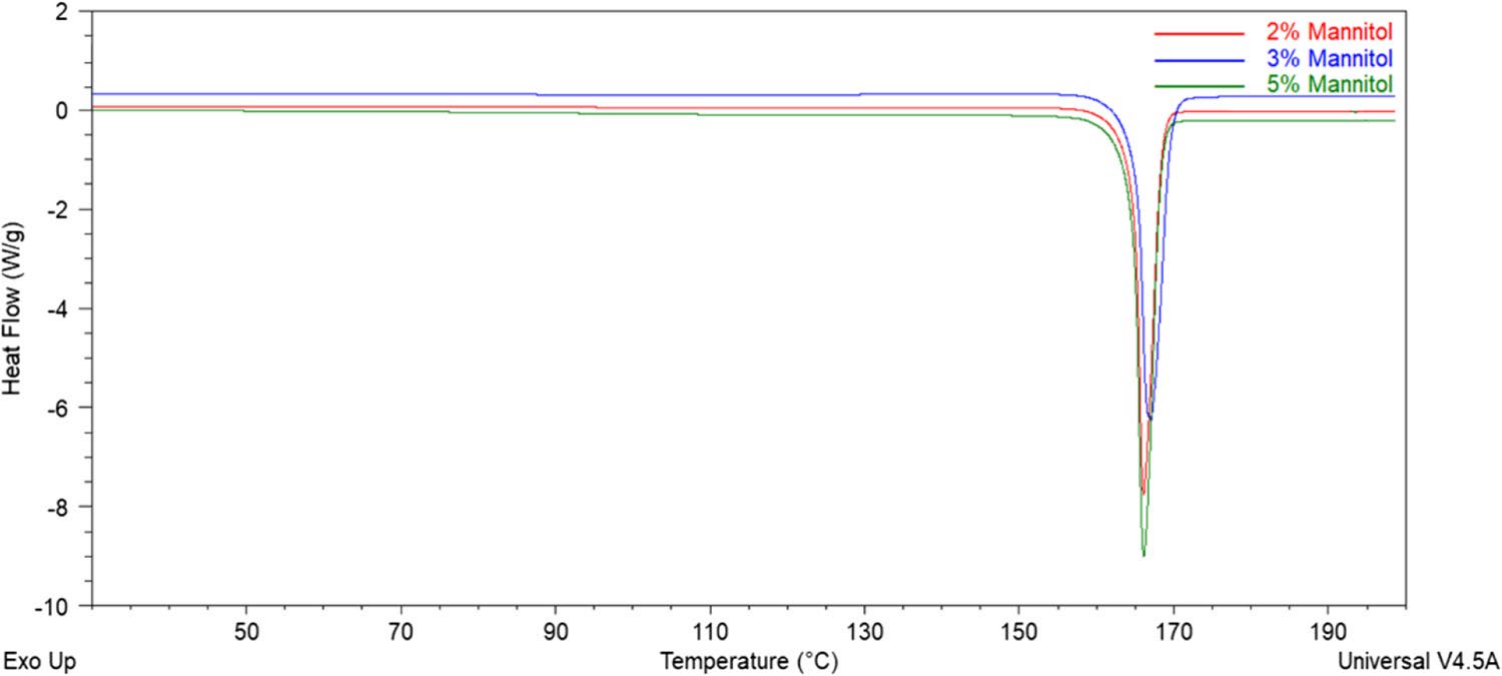
DSC Thermogram of Spray-Dried RALA/pEGFP-N1 NPs. Spray-Dried RALA/pEGFP-N1 NPs were Sealed into a Hermetic Aluminium Pan, then Analysed using DSC Q50 with a Heat Rate of 5ºC/min from 25 to 200ºC. The Thermal Analysis Data were Analysed using Universal Analysis

To determine whether the spray-dried RALA/pEGFP-N1 NPs is amorphous or crystalline, DSC analysis was performed. DSC thermograms of the spray-dried NPs demonstrating the characteristic endothermic peak at approximately 168ºC was found (Fig. 5). This shows that the spray-dried powders at different mannitol concentrations were crystalline and the sharp endothermic peak observed corresponds to the melting point of mannitol [23]. Spray-dried mannitol with different NPs vectors, cargo, and process parameters also displayed similar DSC profiles [23, 31, 46]. For instance, a similar DSC thermogram was reported by Chow et al*.*, whereby naked siRNA was spray-dried with mannitol and leucine as excipients and an inlet temperature of 80ºC [23]. Mannitol crystallisation upon drying process typically reduces the ability to protect NPs from dehydration stress [21]. Such crystallisation can be retarded by the addition of particular compounds, including sodium chloride and sodium phosphate [46]. However, the addition of these compounds can also induce NP destabilisation, depending on the NP characteristics [16].

### *In Vitro* Study

#### Transfection Efficiency

Having determined that spray-dried NPs demonstrated the appropriate characteristics for efficient cellular uptake, *in vitro* transfections were then evaluated in A549 cells using fluorescent microscopy and flow cytometric analysis.

**Fig. 6:**
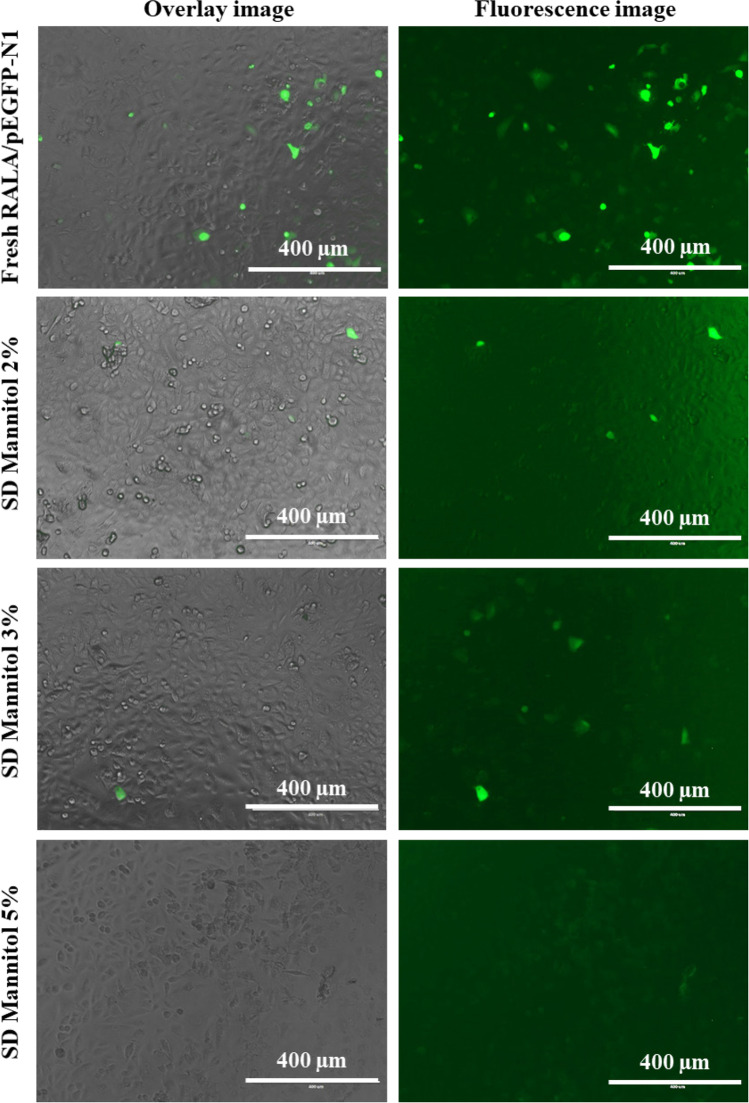
Overlay and fluorescence Image of *in vitro* Transfection Efficiency of Spray-Dried RALA/pEGFP-N1 NPs at Different Mannitol Concentrations in Opti-MEM Media Assessed in A549 cells. Spray-dried RALA/pEGFP-N1 complexes were reconstituted with 50 µL of DNase/RNase-free water and incubated for 3 h at room temperature prior to A549 cells transfection (density of 10^4^ cells per well) for 4–6 h in Opti-MEM media. Subsequently, cells were observed under an optical microscope after transfection. Experiments were performed as three independent replicates, and a representative image is shown for each cryoprotectant

To visually evaluate GFP expression, transfected A549 cells were observed under an optical microscope 48 h post-transfection. The green fluorescence of cells treated with spray-dried RALA/pEGFP-N1 NPs was higher than those treated with freshly prepared NPs (Fig. 6).

**Fig. 7:**
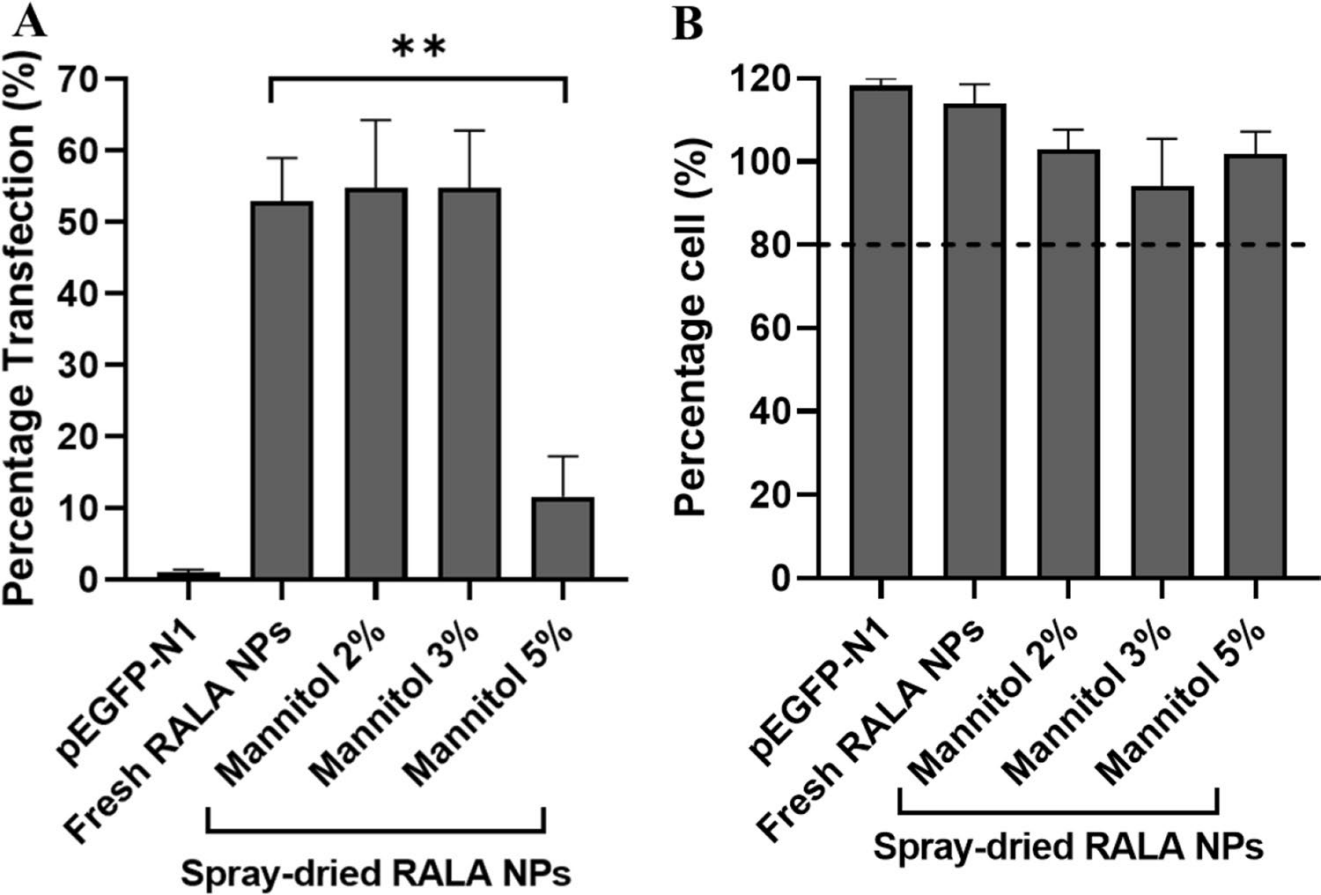
**A** *In-vitro* Transfection of Spray-Dried RALA/pEGFP-N1 complexes at different mannitol concentrations. Cells were trypsinised 48 h and transfection efficiency was measured by flow cytometry. **B** A549 cells viability after 48 h transfection with spray-dried RALA/pEGFP-N1 NPs in Opti-MEM. Spray-dried RALA/pEGFP-N1 complexes were reconstituted with 50 µL of DNase/RNase-free water and incubated for 3 h at room temperature prior to A549 cells transfection (density of 1 × 10^4^ cells per well) for 4–6 h in Opti-MEM media. 48 h post-transfection, cells were incubated with 10% Alamar blue reagent for 2–3 h and absorbance was measured in a plate reader. Results displayed as mean ± SEM, n = 3. (** p < 0.01, **** p < 0.0001; ANOVA)

To evaluate the functionality of spray-dried RALA/pEGFP-N1 NPs, the transfection efficiency in A549 cells was measured. The transfection efficiency of spray-dried RALA/pEGFP-N1 NPs with 2–3% mannitol was approximately 55% and was not significantly different to that of freshly prepared NPs at 53% (Fig. 7A). However, the transfection efficiency of spray-dried RALA/pEGFP-N1 NPs with 5% mannitol was significantly lower than other spray-dried NPs and the freshly prepared NPs (p < 0.01). The retained functionality of spray-dried RALA/pEGFP-N1 NPs with 2–3% mannitol is comparable to the study by Qiu et al*.* with spray-dried PEGylated KL4/mRNA, where 1.5% mannitol was used [18]. With similar mannitol concentration (1–1.5%), different findings was reported by Liang et al., where the functionality of spray-dried CPPs-based NPs was higher than the freshly prepared NPs. Liang et al*.* suggested that hyperosmolar mannitol solution loosens the cellular tight junction, enhancing the penetration of NPs across the cellular membrane [19, 20]. The effect of hyperosmotic mannitol (> 450 mOsm) on the enhancement of cellular uptake in airway epithelial cell lines (16HBE14o-) was also reported by Nilsson et al. [55]. The phosphorylation of caveolin-1 inducing a caveolae-mediated endocytosis pathway also explains the enhanced cellular uptake by mannitol solution [56, 57]. Considering these findings, an enhanced cellular uptake should also have occurred in our study. However, the significant increase of Z-average after spray drying (66 nm to 160 nm) eventually conceal the mannitol effect, resulting in a negligible difference in transfection efficiency between the freshly prepared and spray-dried NPs. It has been reported that RALA/pEGFP-N1 NPs penetrate cellular membrane via clathrin- or caveolin-mediated pathway [5], allowing the NPs size as the essential parameter in the cellular uptake [39, 58]. For example, Gessner et al*.* reported that CPPs-functionalised silica NPs at a size of 50 nm demonstrated significantly higher transfection efficiency in HeLa cells (12,000 fluorescence intensity/a.u.) than 150 and 300 nm NPs (< 6,000 fluorescence intensity/a.u.) [58].

The significantly lower transfection efficiency in spray-dried RALA/pEGFP-N1 NPs in 5% mannitol is probably due to the cytotoxicity effect of a higher concentration of mannitol. This suggestion is supported by Feng et al*.*, who reported the reduction of 43 ± 2% of endothelial cells viability, 30 min post-incubation with 1200 mOsm of mannitol [59]. However, the cell viability study (Fig. 7B) revealed that spray-dried RALA/pEGFP-N1 NPs with up to 5% mannitol were non-toxic, which probably owing to a lower osmolarity than that reported by Feng et al. The functionality reduction in 5% mannitol is most likely due to the higher sugar concentration reducing the cellular uptake. Although Nilsson et al. suggested that 450–700 mOsm mannitol increased the cellular uptake in 16HBE14o- cells [55], higher concentrations and different cell types can provide different results. Huang et al*.* reported the reduction of A549 cellular uptake of fluorescein isothiocyanate chitosan NPs in the presence of 0.45 M sucrose owing to the inhibition of the clathrin-mediated endocytosis pathway [50]. In the transfection study, 5% mannitol of the spray drying formula equates to 0.92 M mannitol in the culture medium, which is higher than the sugar concentration reported by Huang et al*.* This suggests that 5% mannitol can reduce the cellular uptake of RALA/pEGFP-N1 NPs by inhibiting the clathrin-mediated pathway. Furthermore, the use of a higher concentration of mannitol can reduce the respirable fraction, probably due to particle aggregation [60]. The use of a relatively low mannitol concentration is supported by other studies, where the majority of the spray drying of nucleic acid-carrying NPs use a mannitol concentration of 1–3%. The use of a higher concentration of mannitol (10%) was reported by Keil et al. to prepare spray-dried polyethyleneimine (PEI)/pEGFP NPs. Although the Z-average of spray-dried NPs was maintained, the *in vitro* transfection study could not be performed due to the extremely low solubility of the powder [61]. The higher concentration of mannitol (10–12.5%) is typically used for the preparation of more stable active pharmaceutical ingredients, such as salbutamol and doxorubicin [62, 63]. Nevertheless, further studies are important to evaluate the effect of mannitol concentration on cellular uptake and gene expression in different cell and nucleic acid types.

#### Cell Viability

To evaluate the cytotoxicity of spray-dried RALA/pEGFP-N1 NPs, the cell viability of the transfected A549 cells was assessed using the Alamar blue assay. Figure 7B shows that the cell viability of spray-dried RALA/pEGFP-N1 NPs with different mannitol concentrations was > 80%. There was no significant difference between spray-dried and freshly prepared RALA/pEGFP-N1 NPs, and the cell viability of all spray dried NPs was > 80%. Based on ISO 10993–5, these findings suggest that the spray-dried NPs were non-cytotoxic. The safety of RALA condensing different cargo types has previously been reported [3, 4]. However, the toxicity effect can appear after RALA NPs is prepared in any formulation due to the addition of different types of excipient. In the spray drying formula for RALA/pEGFP-N1 NPs, mannitol is the only excipient used, and it has been authorised by Food and Drug Administration (FDA) for pharmaceutical excipients. Furthermore, In the European Union, inhalable mannitol has been approved for the treatment of cystic fibrosis in adults, and in Australia, it has also been approved for children and adults over 6 years [64]. However, safety concerns might still arise since hyperosmolar mannitol (600 mOsm) has been reported to induce embolisation in human umbilical vein endothelial cells (HUVECs), leading to cell death [59]. Shi et al*.* also found that after 48 h of treatment with 250 mmol/L mannitol (equivalent to 4.5% mannitol in the cell culture), the cytoskeleton in HK-2 cells had been damaged [65]. The *in vitro* transfection study with spray-dried RALA NPs in the highest mannitol concentration (5%) provided 2.75% mannitol in the cell culture, which is lower than the toxic concentration found by Shi et al*.*

## CONCLUSION

In this study, we have engineered inhalable powder form of RALA/pEGFP-N1 NPs using nano-spray drying following identification of important experimental variables using factorial design. Results showed that mannitol concentration was the most important variable affecting all responses except for encapsulation efficiency. All measured responses except for encapsulation efficiency demonstrated strong dependency on the experimental variables. The DoE optimised inhalable formulation of RALA/pEGFP-N1 NPs was physicochemically stable, biologically functional with powder properties optimal for lung disease treatment via pulmonary delivery. Hence, the optimal inhalable powder form of RALA NPs with maintained functionality and desirable powder properties represents a robust formulation suitable for developing gene therapy for lung disease treatment. To further develop gene therapy via pulmonary delivery, therapeutic nucleic acids (e.g. miRNA and siRNA) are required to treat different lung diseases. Further investigations are also essential to evaluate and optimise the aerosol performance of the dry powder formulations, the mucus penetration ability mimicking powder behaviour inside the human lung and the *in vivo* profile of the dry powder formulations in preclinical animal models delivered via inhalation.
